# Assessment of Small Vessel Function Using 7T MRI in Patients With Sporadic Cerebral Small Vessel Disease: The ZOOM@SVDs Study

**DOI:** 10.1212/WNL.0000000000209136

**Published:** 2024-02-05

**Authors:** Hilde Van Den Brink, Stanley Pham, Jeroen C. Siero, Tine Arts, Laurien Onkenhout, Hugo Kuijf, Jeroen Hendrikse, Joanna M. Wardlaw, Martin Dichgans, Jaco J. Zwanenburg, Geert Jan Biessels

**Affiliations:** From the Department of Neurology and Neurosurgery (H.V.D.B., L.O., G.J.B.), UMC Utrecht Brain Center; Department of Radiology (S.P., J.C.S., T.A., J.H., J.J.Z.), Center for Image Sciences, University Medical Center Utrecht; Spinoza Centre for Neuroimaging Amsterdam (J.C.S.); Image Sciences Institute (H.K.), University Medical Center Utrecht, the Netherlands; Brain Research Imaging Centre (J.M.W.), Centre for Clinical Brain Sciences, UK Dementia Research Institute Centre at the University of Edinburgh, United Kingdom; Institute for Stroke and Dementia Research (M.D.), University Hospital, LMU Munich; Munich Cluster for Systems Neurology (SyNergy) (M.D.); and German Center for Neurodegenerative Disease (DZNE) (M.D.), Germany.

## Abstract

**Background and Objectives:**

Cerebral small vessel disease (cSVD) is a major cause of stroke and dementia, but little is known about disease mechanisms at the level of the small vessels. 7T-MRI allows assessing small vessel function in vivo in different vessel populations. We hypothesized that multiple aspects of small vessel function are altered in patients with cSVD and that these abnormalities relate to disease burden.

**Methods:**

Patients and controls participated in a prospective observational cohort study, the ZOOM@SVDs study. Small vessel function measures on 7T-MRI included perforating artery blood flow velocity and pulsatility index in the basal ganglia and centrum semiovale, vascular reactivity to visual stimulation in the occipital cortex, and reactivity to hypercapnia in the gray and white matter. Lesion load on 3T-MRI and cognitive function were used to assess disease burden.

**Results:**

Forty-six patients with sporadic cSVD (mean age ± SD 65 ± 9 years) and 22 matched controls (64 ± 7 years) participated in the ZOOM@SVDs study. Compared with controls, patients had increased pulsatility index (mean difference 0.09, *p* = 0.01) but similar blood flow velocity in basal ganglia perforating arteries and similar flow velocity and pulsatility index in centrum semiovale perforating arteries. The duration of the vascular response to brief visual stimulation in the occipital cortex was shorter in patients than in controls (mean difference −0.63 seconds, *p* = 0.02), whereas reactivity to hypercapnia was not significantly affected in the gray and total white matter. Among patients, reactivity to hypercapnia was lower in white matter hyperintensities compared with normal-appearing white matter (blood-oxygen–level dependent mean difference 0.35%, *p* = 0.001). Blood flow velocity and pulsatility index in basal ganglia perforating arteries and reactivity to brief visual stimulation correlated with disease burden.

**Discussion:**

We observed abnormalities in several aspects of small vessel function in patients with cSVD indicative of regionally increased arteriolar stiffness and decreased reactivity. Worse small vessel function also correlated with increased disease burden. These functional measures provide new mechanistic markers of sporadic cSVD.

## Introduction

Cerebral small vessel disease (cSVD) is a disorder of the small perforating arteries, capillaries, and venules in the brain and a major cause of stroke and dementia in the elderly population.^1^ A challenge for research into the underlying disease mechanisms is that the diameter of the affected blood vessels is so small that they are difficult to investigate in vivo in humans. Therefore, previous studies of these vessels in cSVD mostly involved postmortem neuropathology, observing loss of smooth muscle cells, fibrosis, thickening of the vessel walls, and luminal narrowing or even occlusion in cerebral arterioles, but also abnormalities in capillaries and venules.^1^ These structural alterations of the vessels are thought to affect their function, which was indeed observed in experimental animal models of small vessel disease.^2,3^ Measuring small vessel function in humans could provide insight into possible disease mechanisms at the level of the small vessels and thus contribute to the development of urgently needed treatment. Standards for Reporting Vascular Changes on Neuroimaging 2 (STRIVE-2) identified such measures as emerging markers of cSVD.^4^

Vessel function on MRI in patients with sporadic cSVD has been the topic of previous (3T-MRI) studies, mostly involving assessment of perfusion, blood-brain barrier permeability, and vascular reactivity to vasodilating effects of hypercapnia or acetazolamide as well as blood flow pulsatility in large intracranial arteries.^5-12^ Although the findings from these studies vary, an emerging observation is that vessel function decreases with increasing white matter hyperintensity (WMH) burden.^5-12^ With technical advancements in 7T-MRI, we can now study complementary aspects of small vessel function in several vessel populations with a sensitivity and temporal and spatial resolution that was not possible before. These measures can inform on perforating artery flow velocity and stiffness and endothelium-dependent and endothelium-independent vascular reactivity in different brain regions.^13^ We have recently shown abnormal small vessel function in multiple vessel populations on these 7T-MRI measures in patients with monogenic cSVD.^14^ Moreover, one small previous study suggests that perforating artery stiffness may be abnormal in patients with sporadic cSVD.^15^

In this study, we used novel complementary 7T-MRI measures to explore which aspects of small vessel function are abnormal in patients with sporadic cSVD and whether small vessel function relates to disease burden.

## Methods

### Study Design and Participants

Patients with symptomatic sporadic cSVD and age-matched and sex-matched controls were included through the ZOOM@SVDs study at the UMC Utrecht hospital in the Netherlands between March 2017 and April 2021.^13^

- A total of 54 patients were recruited from the stroke, rehabilitation, and memory clinics of the UMC Utrecht and referring neighboring clinics. Sporadic cSVD was defined as either having a history of a clinical lacunar stroke in the past 5 years with a compatible small subcortical infarct visible on MRI or CT or objective cognitive impairment based on a cognitive measurement tool (e.g., CAMCOG) with Fazekas ≥ 2 WMH on MRI. Patients included with a clinical lacunar stroke could not have cerebral large vessel disease or a major cardioembolic source of embolism. Patients could have no other known neurologic condition affecting the brain, including intracranial vascular malformations.
- Controls (N = 28) were recruited among partners or relatives of the patients and through flyer advertisements within the UMC Utrecht. Controls were age-matched and sex-matched to the patients on a group level. Controls could not have a history of stroke, transient ischemic attacks or cognitive complaints, and “silent” cSVD on 3T brain MRI defined as confluent WMH (Fazekas ≥ 2) or lacunes.

**Figure 1:**
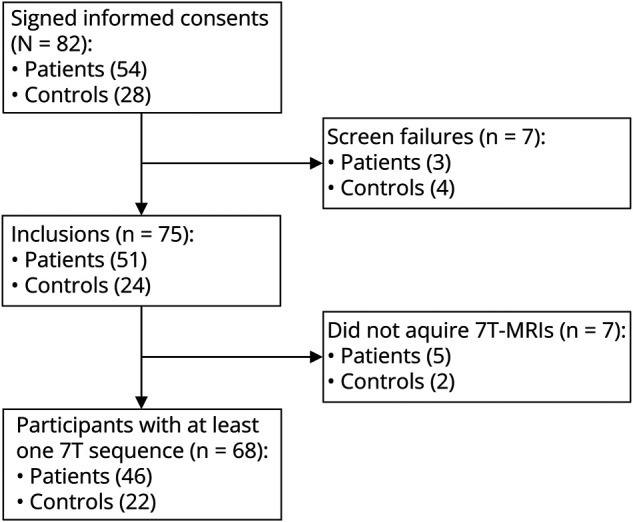
Flowchart of Participant Inclusions and Exclusions Reasons for screen failures are no lacune lesion on study MRI for 3 patients recruited because of a clinical diagnosis lacunar infarct, objective cognitive deficits for one control, an occlusion of the internal carotid on study MRI for one control, and 2 controls who had signs of cSVD on study MRI. Reasons for not acquired 7T-MRIs are scanning was too much a burden for 4 patients and one patient and 2 controls were claustrophobic in the 7T-MRI.

Participants underwent extensive clinical assessment, 3T brain MRI, and 7T brain MRI. Seven participants turned out to be screen failures, and 7 participants could not successfully undergo 7T brain MRI (see Figure 1 for the inclusion and exclusion flowchart), leaving 46 patients and 22 controls for inclusion. Detailed study procedures are published elsewhere.^13^

### Standard Protocol Approvals, Registrations, and Patient Consents

The Medical Ethics Review Committee of the UMC Utrecht (Project number NL62090.041.17) approved the study. Written informed consent was obtained from all participants before any study procedures. The study was registered in the Netherlands Trial Register, NTR6265.

### Clinical Assessment

For patients, stroke, hypertension, hypercholesterolemia, and diabetes mellitus were recorded based on their clinical assessment, and smoking was based on self-report. For controls, these vascular risk factors were based on self-report. Current systolic and diastolic blood pressure and pulse wave velocity were based on 7-day measurements done 3 times a day at home with a telemetric blood pressure device (Tel-O-Graph GSM Plus, graded A/A by the British Hypertension Society).^12^ Participants brought their current medication list to record medication use. All participants underwent a standardized neuropsychological test battery (full test battery reported in design paper^13^). Individual test scores were standardized into *z*-scores using the control group as a reference. Test *z*-scores were then combined to represent specific cognitive domains. The executive function domain is composed of *z*-scores on category fluency, phonemic fluency, trail-making test (TMT) part B/A, and digit span backward. The attention and processing speed domain is composed of *z*-scores on TMT A and digit span forward. A compound score of executive function and attention and processing speed was calculated as the average of the 2 domain *z*-scores.^13^

### 3T Brain MRI

Participants underwent 3T brain MRI on a Philips Achieva 3T scanner with an 8-channel SENSE head coil. The scan protocol included 3D T1-weighted gradient echo, 3D T2-weighted turbo spin echo, 3D T2*-weighted gradient echo, and 3D fluid-attenuated inversion recovery (FLAIR) scan (sequence details eTable 1, links.lww.com/WNL/D397). Lacunes (on T1-weighted, T2-weighted, and FLAIR scan) were manually counted, and microbleed counting (on T2*-weighted scans) was supported by semiautomated rating software.^16^ Ratings were performed by 2 trained raters (HvdB and NAW who had a good interrater reliability of 0.8 or more and resolved discrepancies in a consensus meeting) according to the STRIVE-1 criteria^17^ (note: the criteria for these particular lesions are the same in the updated STRIVE-2).^18^ Segmentations of WMH, lacunes, intracranial volume, and total brain volume were acquired as previously published.^13^

### 7T Brain MRI

Participants underwent 7T brain MRI on a Philips 7T scanner with a 32-channel receive head coil with a quadrature transmit coil (Nova Medical, MA). The scan protocol included 2D-Qflow velocity mapping acquisitions and blood-oxygen–level dependent (BOLD) sequences (sequence details eTable 1, links.lww.com/WNL/D397), with which 3 complementary aspects of small vessel function were measured.

#### Perforating Artery Flow Velocity and Pulsatility Index

At the level of the centrum semiovale and basal ganglia, 2D-Qflow velocity mapping acquisitions were used to assess flow velocity in perforating arteries. A peripheral pulse unit was used for retrospective cardiac gating. Blood flow pulsatility was calculated as (Vmax-Vmin)/Vmean, where Vmax, Vmin, and Vmean are the maximum, minimum, and mean of the normalized and averaged blood flow velocity over the cardiac cycle, respectively. Mean blood flow velocity and pulsatility index within perforating arteries were calculated as the main outcome measures (see eAppendix 1, links.lww.com/WNL/D397 for the reasoning in choosing the mean vs median). Pulsatility index provides—among others—an indication of perforating artery stiffness.

#### Small Vessel Reactivity to a Brief Visual Stimulus

Neurovascular coupling-dependent vascular reactivity in the visual cortex was assessed by estimating the average BOLD hemodynamic response function (HRF) to a brief (500 ms) visual stimulus. The region of interest was subject-specific and was based on the activation pattern in response to long blocks (16.7 seconds) of visual stimulation. The BOLD signal on 7T-MRI is weighted more toward signals from the microvasculature (e.g., capillaries, small venules) than on lower field strength,^19^ which allows us to measure BOLD reactivity in small vessels as long as the large (pial) veins are removed from the region of interest. In addition, the high temporal resolution on 7T-MRI provides a precise estimate of the HRF, from which the peak BOLD% signal change and full width at half maximum were derived as the main outcome measures.

#### Whole-Brain Small Vessel Reactivity to a Hypercapnic Stimulus

Endothelial-independent whole-brain vascular reactivity to hypercapnia (i.e., breathing 6% CO_2_ in air for 2 x 2 minutes) was measured with the BOLD response. Monitoring equipment recorded pulse rate and end-tidal CO_2_ (40 Hz, CD3-A AEI Technologies, Pittsburgh) (based on Thrippleton et al.,^20^ as specified in the design paper^13^). The signal from large (pial) veins was also excluded, so the remaining BOLD signal primarily represents small vessel reactivity. The high spatial resolution of BOLD on 7T-MRI permits regional analyses. The BOLD% signal change in the cortical gray matter, subcortical gray matter, white matter, and WMH were derived as the main outcome measures.

The order of MRI scans was strictly adhered to. After the first BOLD scan with visual stimulation, every participant was taken out of the scanner for a short break. Then, the participant puts on the breathing mask, and the protocol was continued with the hypercapnic challenge. The details on data processing pipelines were published before.^14^ The researchers were blinded for participant group when analyzing these data.

### Statistical Analyses

Differences in characteristics between patients with cSVD and controls were tested with independent samples t tests for continuous normally distributed data, Mann-Whitney *U* tests for nonnormally distributed data, and χ^2^ for categorical data.

Because measures of small vessel function on 7T-MRI are relatively new, we explored whether age and sex are possible confounders in both groups separately, as well as possible relations between antiplatelet, statin, and antihypertensive medication use with small vessel function measures in the patient group. Considering the available sample size, we did not perform more elaborate evaluation on other biomedical factors (e.g., vascular risk factors) and small vessel function.

Linear regression was used to test the relation between measures of small vessel function with age, and independent samples t tests were used to test for a relation between measures of small vessel function and sex (see eTable 2, links.lww.com/WNL/D397). Based on these explorative findings, differences between patients and controls in small vessel function measures were tested with ANCOVA, with age and sex as covariates. Pulsatility index was additionally corrected for mean blood flow velocity, and BOLD hypercapnia comparisons were additionally corrected for change in end-tidal CO_2_ in response to hypercapnia. Within the patients, BOLD% signal change to hypercapnia was compared in normal-appearing white matter (NAWM) and WMH with a paired *t* test.

In secondary analyses, linear regression models were used to see whether measures of small vessel function that differed between patients and controls were associated with indicators of disease burden, defined as cognitive function, WMH volume, and lacune count. Blood flow velocity and pulsatility were considered together in these analyses, because of their close interrelation. WMH volume and lacune count were cube-root transformed because data were skewed, and WMH volume was additionally normalized to the intracranial volume. The linear regression models were corrected for age and sex, and the association with cognitive function was additionally corrected for education. The standardized betas are reported for ease of comparison between variables. To test whether the associations were unique for patients (and thus likely pathological), we tested the significant associations in control participants as well.

All statistical analyses were performed in SPSS version 25, and *p* < 0.05 was considered significant.

### Data Availability

Data on the ZOOM@SVDs study used in these analyses that are not published within this article are available by request from any qualified investigator.

## Results

Characteristics of patients with cSVD and controls are shown in Table 1. Patients and controls were well matched for age and sex. As expected, patients more often had hypertension and hypercholesterolemia and more often used antihypertensives, statins, and antiplatelet drugs. Characteristic cSVD imaging markers were also more prominent in patients than controls (details in Table 1).

**Table 1:** Characteristics of Patients With Sporadic cSVD and Controls

	Sporadic cSVD (n = 46)	Control (n = 22)	*p* Value
Demographics			
Age, M ± SD	65.3 ± 9.4	63.5 ± 6.6	0.35
Female sex, n (%)	15 (33)	8 (36)	0.79
Cognition and depression			
Executive function, attention and processing speed, *z*-score	−0.74 ± 0.77	0.12 ± 0.58	<0.001
CES-D	11 ± 8	6 ± 4	<0.001
Vascular risk profile			
Stroke, n (%)	31 (67)	0 (0)	<0.001
Hypertension, n (%)	39 (85)	3 (14)	<0.001
Current 7-d systolic BP, M ± SD [mm Hg]	129.7 ± 11.6	126.3 ± 10.7	0.25
Current 7-d diastolic BP, M ± SD [mm Hg]	85.3 ± 7.9	85.8 ± 9.7	0.83
Pulse wave velocity, M ± SD	9.5 ± 1.5	9.2 ± 0.9	0.36
Hypercholesterolemia, n (%)	34 (74)	7 (32)	0.001
Diabetes mellitus^a^, n (%)	7 (15)	0 (0)	0.09
Current/ever smoker, n (%)	38 (83)	14 (64)	0.13
Medication use			
Antihypertensives, n (%)	38 (83)	4 (18)	<0.001
Statins, n (%)	32 (70)	4 (18)	<0.001
Antiplatelet drugs, n (%)	36 (78)	1 (5)	<0.001
3T MRI SVD markers			
WMH volume, median [min-Q1-Q3-max] [% of ICV]	1.03 [0.02–0.34–1.65–4.73]	0.05 [0.00–0.03–1.03–2.77]	<0.001
Lacune presence, n (%)	30 (65)	0 (0)	<0.001
Lacune count^b^, median [Q1–Q3]	3 [1–6]	0 [0-0]	
Microbleed presence, n (%)	23 (50)	4 (18)	0.02
Microbleed count^c^, median [Q1–Q3]	3 [2–12]	2 [1.5–2.5]	0.22
Brain volume, M ± SD [% of ICV]	69.9 ± 6.4	73.5 ± 4.2	0.02

Abbreviations: BP = blood pressure; CES-D = Center for Epidemiologic Studies Depression Scale; ICV = intracranial volume; M = mean; Q1–Q3 = quartile 1 and quartile 3; SD = standard deviation; WMH = white matter hyperintensity.

a All patients with diabetes mellitus were treated with glucose lowering medication.

b Count for participants with ≥ 1 lacune(s).

c Count for participants with ≥ 1 microbleed(s).

**Figure 2:**
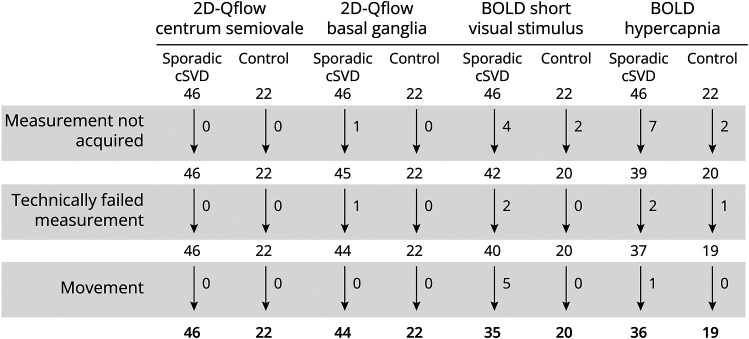
Flowchart Showing Reasons for Excluded Scans per Small Vessel Function Measure Reasons for not acquired measurements are the participant could not continue the scanning session for the 2D-Qflow scan (1); glasses that would not fit in the head coil (3), very bad vision of the participant (2), or unable to finish the sequence (1) for the BOLD short visual stimulus scan; and equipment not yet available (5) or participants unwilling or unable to do or finish the hypercapnia challenge (4) for the BOLD hypercapnia scan.

Figure 2 lists the number of participants for each of the 7T measures included in the analyses. Participants with missing or failed 7T measurements did not differ from participants with complete 7T data sets for demographics or disease severity (all *p* > 0.2).

Within the patient and control groups, explorative analyses showed possible relations of age and sex with small vessel function measures (see eTable 2, links.lww.com/WNL/D397). Specifically, higher age related with lower blood flow velocity in perforating arteries in the basal ganglia in patients, and male sex related with decreased blood flow velocity in perforating arteries in the basal ganglia in patients and increased pulsatility index in these perforating arteries in both patients and controls. The primary and secondary analyses were, therefore, corrected for age and sex.

### Small Vessel Function in cSVD vs Controls

#### Perforating Artery Flow Velocity and Pulsatility Index

**Figure 3:**
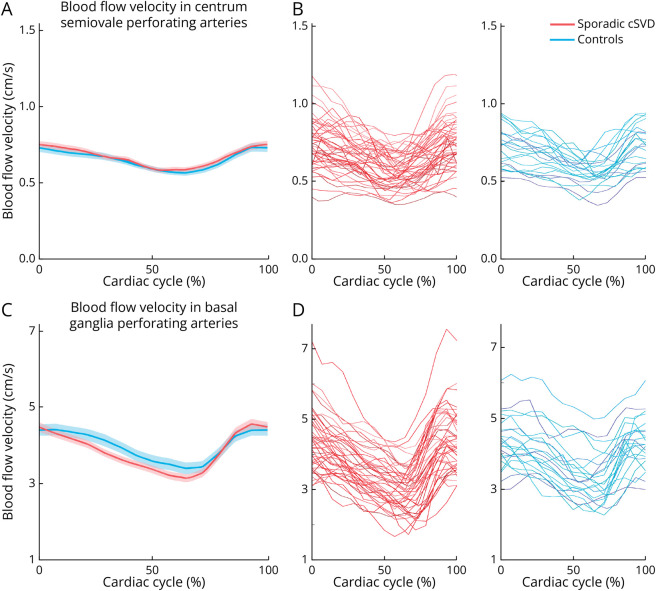
Mean and Individual Participants Blood Flow Velocity Traces The mean blood flow velocity (solid lines) and standard errors of the mean (shaded lines) in perforating arteries in the centrum semiovale (A) and basal ganglia (C) for patients with sporadic cSVD and controls. Individual blood flow velocity traces in perforating arteries in the centrum semiovale (B) and basal ganglia (D) for sporadic cSVD in red and controls in blue.

The number of perforating arteries included in the analyses were similar (i.e., similar density, calculated as the number of perforating arteries per cm^2^ of the subject-specific region of interest) in patients with cSVD (centrum semiovale, median N = 48, density mean ± SD 2.23 ± 1.13/cm^2^; basal ganglia, median N = 19, density mean ± SD 0.91 ± 0.27/cm^2^) and controls (centrum semiovale, median N = 44, density mean ± SD 2.27 ± 0.89/cm^2^, *p* = 0.92; basal ganglia, median N = 19, density mean ± SD 0.92 ± 0.29/cm^2^, *p* = 0.99). Figure 3, A and C display the mean blood flow velocity in perforating arteries in the centrum semiovale and basal ganglia, respectively, in sporadic cSVD and controls (see Figure 3, B and D for individual traces). In perforating arteries in the basal ganglia, pulsatility index was increased in patients vs controls, whereas mean blood flow velocity was similar (Table 2). In perforating arteries in the centrum semiovale, mean blood flow velocity and pulsatility index in the 2 groups were similar (Table 2). The number of perforating arteries that was detected in WMH in patients was too low (median 1, maximum 7 in centrum semiovale) to reliably compare perforating artery blood flow velocity and pulsatility index in NAWM vs WMH.

**Table 2:** Small Vessel Function on 7T-MRI in Patients With Sporadic cSVD and Controls

	Sporadic cSVD	Control	*p* Value
2D-Qflow centrum semiovale^a^	n = 46	n = 22	
Blood flow velocity [cm/s]	0.65 ± 0.12	0.64 ± 0.10	0.70
Pulsatility index^b^	0.35 ± 0.13	0.32 ± 0.11	0.40
2D-Qflow basal ganglia^a^	n = 44	n = 22	
Blood flow velocity [cm/s]	3.73 ± 0.69	3.87 ± 0.68	0.55
Pulsatility index^b^	0.45 ± 0.14	0.36 ± 0.13	0.01
BOLD short visual stimulus^c^	n = 35	n = 20	
Peak BOLD% signal change	0.63 ± 0.20	0.67 ± 0.16	0.46
Full width at half max [s]	3.37 ± 0.97	4.00 ± 0.80	0.02
BOLD hypercapnic stimulus^d^	n = 36	n = 19	
CGM BOLD% signal change	3.72 ± 1.37	3.29 ± 1.51	0.17
SGM BOLD% signal change	3.92 ± 1.45	3.47 ± 1.56	0.30
WM BOLD% signal change	0.58 ± 0.41	0.59 ± 0.35	0.72

Abbreivations: BOLD = blood-oxygen–level dependent; CGM = cortical gray matter; SGM = subcortical gray matter; WM = white matter.

Data are shown as mean ± SD.

a The region of interest is the entire centrum semiovale and basal ganglia excluding lacunes. Statistical analyses included age and sex as covariates.

b Statistical analyses additionally included blood flow velocity as covariate.

c Statistical analyses included age and sex as covariates.

d Statistical analyses included age, sex, and change in end-tidal CO_2_ to hypercapnia as covariates.

#### Small Vessel Reactivity to a Brief Visual Stimulus

**Figure 4:**
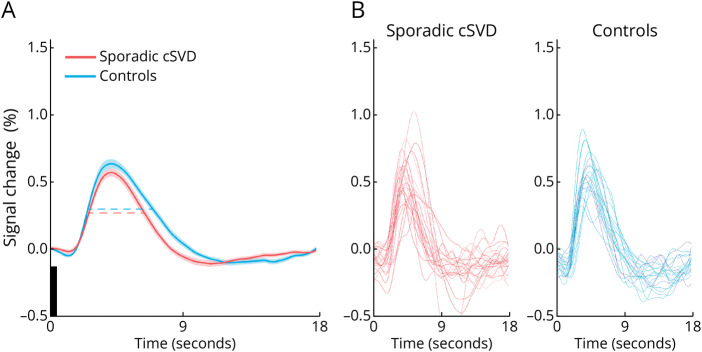
Hemodynamic Response Functions After Visual Stimulation (A) Shown are the average hemodynamic response function estimates (solid lines) and standard errors of the mean (shaded areas) after 500 ms visual stimulation (black bar) for patients with sporadic cSVD (red) and controls (blue). The horizontal dotted lines indicate the full width at half maximum which was significantly shorter in patients (mean ± SD: 3.37 ± 0.97) than in controls (4.00 ± 0.80, *p* = 0.02). (B) Individual BOLD hemodynamic response function estimates after 500 ms visual stimulation for all patients with sporadic cSVD and controls.

Figure 4A shows the estimates of the average hemodynamic response function (HRF) in the visual cortex in response to a brief visual stimulus in patients and controls (Figure 4B provides the individual responses per participant). Of note, the size of the participant-specific regions of interest (i.e., percentage of activated voxels in the imaging region) in which the estimates were made was similar between patients (mean ± SD 25.6 ± 12.5%) and controls (30.4 ± 12.5%, *p* = 0.23). The full width at half-maximum of the HRF was shorter in patients compared with controls, as indicated by the dotted lines in Figure 4A (corresponding numbers are in Table 2). There were no significant group differences in peak BOLD% signal change (Table 2), onset time (patients mean ± SD 1.90 ± 0.50 seconds; controls 1.73 ± 0.68 seconds, *p* = 0.29), and time to peak (patients mean ± SD 4.22 ± 0.70 seconds; controls 4.31 ± 0.78 seconds, *p* = 0.66).

#### Small Vessel Reactivity to a Hypercapnic Stimulus

In the cortical gray matter, subcortical gray matter, and total white matter, BOLD% signal change to hypercapnia was similar in patients and controls (Table 2). In patients, BOLD% signal change was lower in WMH (0.31 ± 0.34) than in NAWM (0.65 ± 0.52, *p* < 0.001). These analyses were based on 29 patients because in 7 patients, the volume of WMH was too small (arbitrary cutoff at <0.3% of intracranial volume) for regional vascular reactivity assessment.

### Small Vessel Function and Disease Burden in Patients With Sporadic cSVD

In age and sex-corrected linear regression models, measures of small vessel function were associated with several indicators of disease burden. Specifically, decreased blood flow velocity was significantly associated with worse executive function, attention and processing speed, higher WMH volume, and higher lacune count (Table 3). Increased pulsatility index was associated with a higher lacune count (Table 3). Finally, a shorter full width at half maximum of the BOLD response to a brief visual stimulus was associated with worse cognition and higher WMH volume (Table 3). Of note, these associations were not observed in control participants (eTable 3, links.lww.com/WNL/D397). In the patient group, there was no relation between small vessel function measures and antiplatelet, statin, or antihypertensive drug use (all *p* > 0.06).

**Table 3:** Associations of Small Vessel Dysfunction With Cognition, White Matter Hyperintensity Volume, and Lacune Count in Patients With Sporadic cSVD

	Cognition^b^	WMH volume	Lacune count
2D-Qflow basal ganglia^a^			
Blood flow velocity [cm/s]	β = 0.38	β = −0.38	β = −0.39
	CI = 0.03 to 0.73	CI = −0.69 to −0.07	CI = −0.70 to −0.07
	*p =* 0.04	*p =* 0.02	*p =* 0.02
Pulsatility index	β = −0.24	β = 0.08	β = 0.36
	CI = −0.63 to 0.16	CI = −0.25 to 0.41	CI = 0.04 to 0.68
	*p =* 0.24	*p =* 0.64	*p =* 0.03
BOLD short visual stimulus			
Full width at half maximum [s]	β = 0.39	β = −0.49	β = 0.06
	CI = 0.06 to 0.73	CI = −0.75 to −0.21	CI = −0.29 to 0.41
	*p =* 0.02	*p =* 0.001	*p =* 0.74
BOLD hypercapnic stimulus			
WMH BOLD% signal change	β = 0.08	β = −0.05	β = 0.18
	CI = −0.44 to 0.60	CI = −0.43 to 0.35	CI = −0.20 to 0.06
	*p =* 0.76	*p =* 0.82	*p =* 0.34

Abbreviations: β = standardized beta; BOLD = blood-oxygen–level dependent; CI = 95% confidence interval; CGM = cortical gray matter; WMH = white matter hyperintensity.

Tested with linear regressions, with an age and sex correction and standardized betas, are reported for ease of comparison between variables. We analyzed the relation for both blood flow velocity and pulsatility index because the 2 are so strongly interrelated. WMH volume was normalized for intracranial volume and cube-root transformed, and lacune count was cube-root transformed.

a The region of interest is the entire basal ganglia minus lacunes.

b Compound score of executive function and attention and processing speed. This analysis was corrected for age, sex, and education and in additional analyses also for CES-D score (as a measure of depressive symptoms). The latter did not change the reported results (data not shown).

## Discussion

Using cutting-edge techniques on 7T-MRI, we found abnormalities in cerebral small vessel function in patients with sporadic cSVD compared with age-matched and sex-matched controls, including increased pulsatility index in perforating arteries in the basal ganglia, shorter vascular reactivity to brief visual stimulation in the occipital cortex, and decreased reactivity to hypercapnia within WMH. Among patients, blood flow velocity and pulsatility index in basal ganglia perforating arteries and reactivity to brief visual stimulation related to disease burden. These findings likely reflect regionally increased arteriolar stiffness and decreased endothelial-dependent and endothelial-independent vascular reactivity, which become increasingly affected with progression of cSVD disease severity.

Previous studies on pulsatility index of the cerebral vasculature mostly studied the internal carotid and middle cerebral arteries, where a relationship was observed between increased pulsatility of flow in these vessels and WMH volume.^11^ Here, we assessed pulsatility at the level of the perforating arteries. We observed increased pulsatility index in small perforating arteries within the basal ganglia in patients compared with controls, also in relation to higher disease burden. These findings seem in accordance with 2 small previous studies in sporadic cSVD. One study also observed increased pulsatility in perforating arteries within the basal ganglia,^15^ whereas the other did not, possibly reflecting the relatively low disease burden in that study.^21^ Interestingly, we found that pulsatility index was unaffected in perforating arteries within the centrum semiovale, whereas we previously reported increased pulsatility index in centrum semiovale perforating arteries in patients with CADASIL and patients with either lacunar infarction or deep intracerebral hemorrhage.^14,15^ These findings imply that underlying pathogenesis may differ in small vessel populations throughout the brain and that these spatial differentiations may depend on the type of cSVD under study. Pulsatility index measured from the velocity trace is known to reflect local vessel stiffness,^22^ but it is important to note that the measured pulsatility index is also dependent on effects upstream and downstream in the vascular tree.^23^ The increased pulsatility index that we observed could therefore reflect a higher pulsatile perfusion pressure transmitted through the larger upstream vessels, enhanced stiffness of the perforating arteries themselves and/or changes in the downstream vascular bed such as abnormal microvascular compliance. Pulse wave velocity in the brachial artery was similar between patients and controls. This, in combination with what we know about the small vessel wall changes in cSVD that are known to lead to stiffened vessel walls and luminal narrowing at the level of arterioles, capillaries, and venules,^1^ makes that we interpret the increased pulsatility index in the present cohort as vessel stiffness of the perforating arteries at the point that we measured or of the downstream smaller arterioles.

Vascular reactivity has previously mostly been studied using 3T-MRI or lower field strength, with several types of stimuli probing different physiologic pathways in different vessel populations. Neuronal task-based approaches, such as motor and visual stimulation, have been used to study endothelial-dependent reactivity based on neurovascular coupling. Hypercapnia or administration of vasodilatory medication has been applied to study endothelial-*in*dependent reactivity. Task-based approaches have so far mostly been applied in patients with cerebral amyloid angiopathy, reporting decreased reactivity in response to 19–40 second visual stimulation in patients compared with controls.^24-28^ In this study, we assessed reactivity (i.e., the HRF response) to a brief (0.5 seconds) visual stimulus. Although the peak amplitude of the HRF estimate was similar for patients and controls, the full width at half maximum of the HRF estimate was shorter in patients (Figure 4A), which was related to a higher disease burden. A shorter full width at half maximum could reflect (a combination of) several underlying physiologic changes.^29^ The most likely possible explanation is disturbed blood pooling in downstream venules and veins, for example, through shorter capillary transit time^30^ and/or stenosis or occlusion of arterioles and/or venules.^1^ This possibly disturbs blood flow then worsens with higher disease severity. To our knowledge, a similar setup with a brief visual stimulus on 7T-MRI has only once been used before in cSVD, in our parallel ZOOM@SVDs study in patients with CADASIL, where we observed a lower peak amplitude of the HRF estimate compared with controls, indicative of less blood flow increase in response to brief visual stimulation,^14^ yet without a shorter full width at half maximum. Apparently, abnormalities in neurovascular response profiles may differ between different forms of cSVD. In our study, we also assessed vascular reactivity to (2 × 2 minutes) hypercapnia. Earlier studies on lower field strength reported a relation between decreased reactivity and WMH burden in cSVD.^5-10,31,32^ The spatial resolution of 7T-MRI helps to better assess vascular reactivity to hypercapnia in specific regions of interest. Between patients and controls, reactivity to hypercapnia was not altered in subcortical gray matter, cortical gray matter or white matter. Within the patient group, however, we observed lower reactivity within WMH than NAWM. In our earlier study in patients with CADASIL, we reported similar findings for all these regions of interest.^14^ Our findings thus suggest that in sporadic cSVD, the endothelium-dependent vascular response to quick, subtle, and local demands in the cortex is altered, whereas longer and global stimulation with hypercapnia seems to generate a normal endothelium-independent vascular response in the cortex, although not in WMH.

Overall these results emphasize the strength of combining the assessment of complementary measures of small vessel function within one cohort. These findings indicate that whether small vessel function is altered, depends on factors such as the specific small vessel population that is assessed, whether vessels are assessed in rest or during reactivity responses, and which type and duration of stimulus are used to trigger vessel reactivity. By assessing multiple aspects of small vessel function throughout different vessel populations in the brain, more precise insights into the underlying disease mechanisms within a cohort can be acquired.

This study also has some limitations. Inherent to the demanding study protocol, the participants recruited in this study were mostly not in the latest disease stages of cSVD, which poses a selection bias. This bias is, however, more likely to underestimate than overestimate the reported disease effects. Similarly, not all recruited participants could successfully finish the full scan protocol (Figure 2). Importantly, however, participants' demographics were similar to those for whom complete scan data sets were available, suggesting that selection bias after recruitment was likely minimal. A further limitation is the inherent difference in medication use between patients and controls. Antihypertensive^4,33^ and statin use^34^ are known to affect cerebral vessel function. However, because of the positive effect of medication on vessel function, this bias is more likely to underestimate than overestimate the disease effects we report here. Moreover, within the patient group, we did not find significant associations between antihypertensive, statin, and antiplatelet use and the 7T measures.^35,36^ In addition to medication, there are more variables related to vascular health that might influence small vessel function, including vascular risk factors. Given the collinearity of vascular risk factors and treatment with being a patient (versus a control participant), this study was not powered to address the role of these factors in the observed between-group differences. This should be a topic of further studies. There are limitations of the 7T-MRI measures too. To avoid prolonged scan times, the acquisition protocol for the Qflow sequences had a relatively low temporal resolution,^37^ which could cause underestimation of the pulsatility index. This, however, should not affect the between-group relative differences. In addition, Qflow sequences can fail to detect small perforating arteries with a very low blood flow velocity. However, because the number of detected perforating arteries and the mean blood flow velocity was similar between patients and controls, this is unlikely to have affected the interpretation of our results. Regarding the BOLD sequences, despite the high field strength, particularly in the white matter, the contrast-to-noise ratio (i.e., sensitivity) of the BOLD signal is modest, which limits sensitivity to disease effects. Given the apparent impact of the nature (i.e., duration, intensity) of the stimulus on the deficits in vascular function observed in cSVD, stimulus protocols also need further standardization. A limitation of the setup of our study is the cross-sectional design. Although we report a relation between small vessel dysfunction and disease severity, longitudinal studies are warranted to test if small vessel function predicts worsening parenchymal damage and cognitive decline. Such questions can be addressed with the 2-year follow-up data that are acquired as part of the ZOOM@SVDs study for which the analyses are currently underway.

In conclusion, we observed that small vessel function is affected in patients with cSVD. Our findings likely reflect regionally increased arteriolar stiffness and decreased endothelial-dependent and endothelial-independent vascular reactivity, which are increasingly affected with higher cSVD disease severity. The results thus provide new biomarkers for cSVD at the level of the small vessels themselves. Longitudinal studies are warranted and underway to see if small vessel dysfunction predicts increasing tissue injury and cognitive decline in cSVD, and further technical and biological validation studies are needed^38^ to further establish these markers as intermediate outcome measures for future cSVD drugs trials.

## Appendix Authors

**Table:**

Name	Location	Contribution
Hilde Van Den Brink, PhD	Department of Neurology and Neurosurgery, UMC Utrecht Brain Center, University Medical Center Utrecht, the Netherlands	Drafting/revision of the manuscript for content, including medical writing for content; major role in the acquisition of data; analysis or interpretation of data
Stanley Pham, MSc	Department of Radiology, Center for Image Sciences, University Medical Center Utrecht, the Netherlands	Analysis or interpretation of data
Jeroen C. Siero, PhD	Department of Radiology, Center for Image Sciences, University Medical Center Utrecht; Spinoza Centre for Neuroimaging Amsterdam, the Netherlands	Major role in the acquisition of data; analysis or interpretation of data
Tine Arts, PhD	Department of Radiology, Center for Image Sciences, University Medical Center Utrecht, the Netherlands	Major role in the acquisition of data; analysis or interpretation of data
Laurien Onkenhout, MD	Department of Neurology and Neurosurgery, UMC Utrecht Brain Center, University Medical Center Utrecht, the Netherlands	Major role in the acquisition of data
Hugo Kuijf, PhD	Image Sciences Institute, University Medical Center Utrecht, the Netherlands	Analysis or interpretation of data
Jeroen Hendrikse, MD, PhD	Department of Radiology, Center for Image Sciences, University Medical Center Utrecht, the Netherlands	Study concept or design
Joanna M. Wardlaw, MD	Brain Research Imaging Centre, Centre for Clinical Brain Sciences, UK Dementia Research Institute Centre at the University of Edinburgh, United Kingdom	Study concept or design
Martin Dichgans, MD	Institute for Stroke and Dementia Research, University Hospital, LMU Munich; Munich Cluster for Systems Neurology (SyNergy); German Center for Neurodegenerative Disease (DZNE), Munich, Germany	Drafting/revision of the manuscript for content, including medical writing for content; study concept or design
Jaco J. Zwanenburg, PhD	Department of Radiology, Center for Image Sciences, University Medical Center Utrecht, the Netherlands	Drafting/revision of the manuscript for content, including medical writing for content; study concept or design; analysis or interpretation of data
Geert Jan Biessels, MD, PhD	Department of Neurology and Neurosurgery, UMC Utrecht Brain Center, University Medical Center Utrecht, the Netherlands	Drafting/revision of the manuscript for content, including medical writing for content; study concept or design; analysis or interpretation of data

## Glossary

BOLD: blood-oxygen–level dependent
cSVD: cerebral small vessel disease
HRF: hemodynamic response function
NAWM: normal-appearing white matter
TMT: trail-making test
WMH: white matter hyperintensity
